# Connexins and the atrioventricular node

**DOI:** 10.1016/j.hrthm.2012.10.020

**Published:** 2013-02

**Authors:** Ian P. Temple, Shin Inada, Halina Dobrzynski, Mark R. Boyett

**Affiliations:** * Institute of Cardiovascular Sciences, University of Manchester, Core Technology Facility, 46 Grafton St, Manchester, UK; † National Cerebral and Cardiovascular Center Research Institute, Laboratory of Biomedical Sciences and Information Management, Fujishiro-dai, Suita, Osaka 565-8565, Japan

**Keywords:** AV, atrioventricular, AVNRT, atrioventricular nodal reentrant tachycardia, CN, compact node, Cx, connexion, I_Na_, Na^+^ current, INE, inferior nodal extension, PB, penetrating bundle, Atrioventricular node, Inferior nodal extension, Left nodal extension, Compact node, His bundle, Dual pathway electrophysiology, Atrioventricular nodal reentrant tachycardia, Connexins

## Abstract

The structure and functioning of the atrioventricular (AV) node has remained mysterious owing to its high degree of complexity. In this review article, we integrate advances in knowledge regarding connexin expression in the AV node. Complex patterning of 4 different connexin isoforms with single channel conductances ranging from ultralow to high explains the dual pathway electrophysiology of the AV node, the presence of 2 nodal extensions, longitudinal dissociation in the penetrating bundle, and, most importantly, how the AV node maintains slow conduction between the atria and the ventricles. It is shown that the complex patterning of connexins is the consequence of the embryonic development of the cardiac conduction system. Finally, it is argued that connexin dysregulation may be responsible for AV node dysfunction.

## Introduction

**Figure 1:**
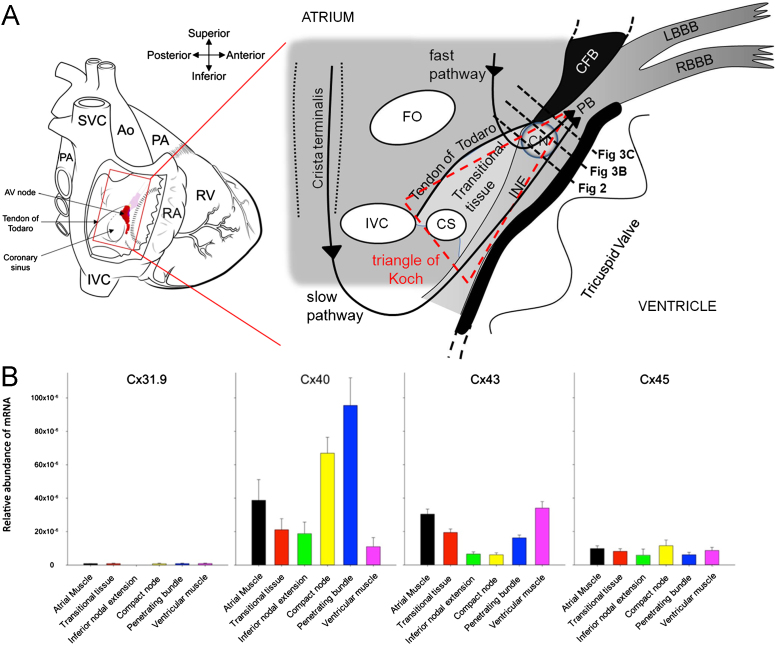
Connexin expression at the mRNA level in different regions of human atrioventricular (AV) junction. **A:** Anatomy of the AV node. *Left:* Heart viewed from behind with window cut in the right atrium to expose the AV node (shown in red). Modified from Li et al.^10^*Right:* Exploded view of boxed region on the left. **B:** Relative abundance of mRNA for 4 connexin isoforms in different regions of the AV junction of the human heart. Means±SEM values are shown (n = 6). From Greener et al.^3^ Ao = aorta; CN = compact node; CS = coronary sinus; FO = fossa ovalis; INE = inferior nodal extension; IVC = inferior vena cava; LBBB = left bundle branch block; PA = pulmonary artery; PB = penetrating bundle; RA = right atrium; RBBB = right bundle branch block; RV = right ventricle; SVC = superior vena cava.

The atrioventricular (AV) node is the “gatekeeper” between the atria and the ventricles and is located at the AV junction on the right side of the heart (Figure 1A). The primary role of the node is to conduct the action potential from the atria to the ventricles. However, the AV node also acts as a backup pacemaker in the case of failure of the sinus node and stops arrhythmias in the atria, such as atrial fibrillation, from being conducted into the ventricles at dangerously high rates. On the other hand, the AV node is part of the circuit underlying AV nodal reentrant tachycardia (AVNRT).^1^ An important aspect of AV node conduction is to introduce a delay between atrial and ventricular excitation to allow time for atrial contraction to complete filling of the ventricles. In the electrocardiogram, the delay between atrial and ventricular excitation corresponds to the PR interval and in humans it is 120–200 ms; in large part, this reflects the slow conduction through the AV node (although the PR interval must also include conduction time across the atrium and through the His-Purkinje system).^2^ The conduction velocity of the slow pathway of the AV node, in rabbits, for example, is 2–10 cm/s, whereas the conduction velocity of the atrial muscle is 80±29 cm/s and the conduction velocity of the Purkinje fibers is 150±20 cm/s.^2–4^ In part, the slow conduction velocity of the AV node is the result of the small diameter of nodal myocytes (conduction velocity is function of cell diameter) and the complex arrangement of the myocytes (nodal myocytes can be separated by extensive connective tissue), which is expected to slow conduction, because the action potential will have to follow a more tortuous path through the AV node. In part, the slow conduction velocity of the AV node is also the result of the poor expression of Na^+^ channels (Na_v_1.5; responsible for Na^+^ current [I_Na_]) in the AV node and the consequent low upstroke velocity of the action potential (conduction velocity is a function of upstroke velocity).^3,5,6^ However, the slow conduction velocity of the AV node is also the result of poor electrical coupling between the myocytes of the AV node. Electrical coupling between cardiac myocytes is provided by gap junctions made of connexins. While some of the story is well known, the detailed examination of connexins at the AV node has revealed some surprising complexities, as explained in this brief review. An expansion of this review (including outline of history, embryology, and anatomy of the AV node) is available as an online supplement.

## Connexin expression within the AV node

**Figure 2:**
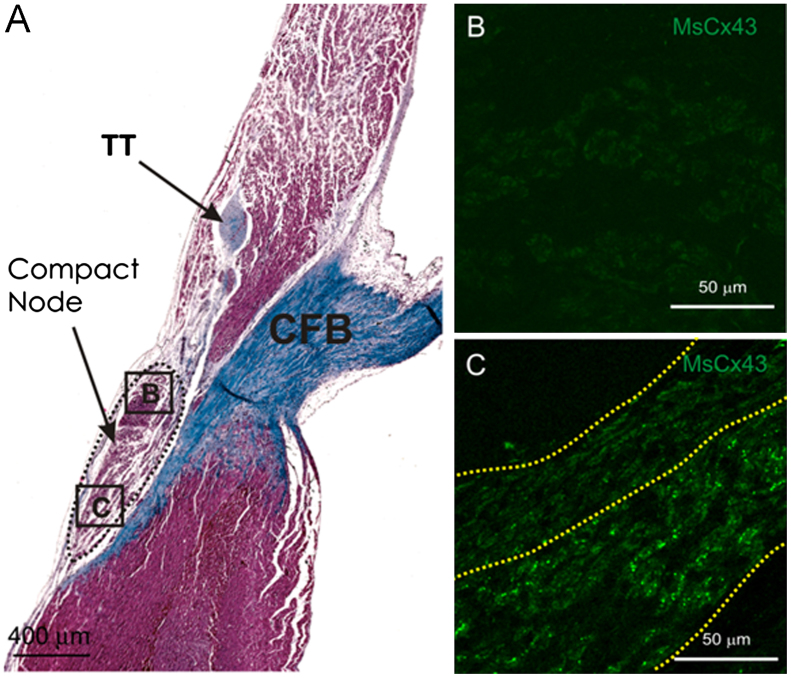
Heterogeneous expression of connexin 43 (Cx43) in the compact node (CN) of the rabbit heart. **A:** Masson’s trichrome stained section through the CN of the rabbit heart (myocytes stained red; connective tissue stained blue). The CN is ringed with a dashed line. **B and C:** High-magnification images of boxed regions in panel A (B = CN; C = lower nodal bundle) showing Cx43 expression (immunofluorescence; bright green punctate spots). In panel C, dotted yellow lines divide tissue into Cx43-negative (top) and Cx43-positive (bottom) regions. Modified from Dobrzynski et al.^12^ CFB = central fibrous body; TT = tendon of Todaro.

**Figure 3:**
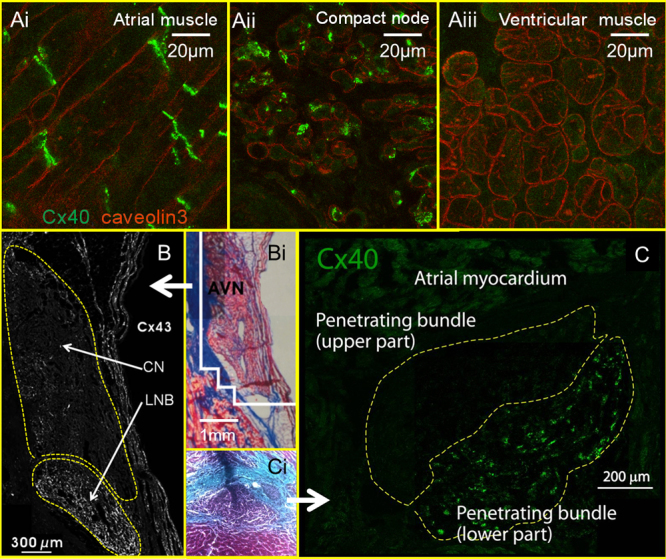
Heterogeneous expression of connexins in atrioventricular (AV) conduction axis. **A:** Expression of connexin 40 (Cx40) (immunofluorescence; green signal) in atrial muscle (Ai), compact node (CN) (Aii), and ventricular muscle (Aiii) of the human heart. Sections are also immunolabeled for caveolin3 (red signal), which is present in the cell membrane of all cardiac myocytes. Cx40 labeling is present at an intercalated disk in atrial myocytes, punctate in the CN, and absent in ventricular myocytes. From H Dobrzynski, unpublished data. **B:** Expression of Cx43 (immunofluorescence; white punctate signal) in the CN of the human heart. CN (ringed in yellow) is Cx43 negative, whereas lower nodal bundle (LNB; also ringed in yellow) is Cx43 positive. Modified from Hucker et al.^11^**Bi:** Section corresponding to that in panel B stained with Masson’s trichrome (myocytes stained red; connective tissue stained blue). **C:** Expression of Cx40 in PB (ringed in yellow) of the rat heart (immunofluorescence; green punctate signal). The upper part of the bundle is Cx40 negative, and the lower part is Cx40 positive. Modified from Yoo et al.^6^**Ci:** Section corresponding to that in panel C stained with Masson’s trichrome.

Four connexin isoforms are expressed in heart: Cx40, which forms large-conductance (200 pS) gap junction channels; Cx43, which forms medium-conductance (60–100 pS) gap junction channels; Cx45, which forms small-conductance (20–40 pS) gap junction channels; and Cx30.2 (or Cx31.9 in humans), which forms ultra-small-conductance (9 pS) gap junction channels.^7^ Cx30.2/Cx31.9 is special in that it will form heterotypic gap junction channels with other connexins, and the heterotypic channels have a small conductance (15–18 pS).^7^ Electrical coupling in the AV node is poor because there are few gap junctions between the nodal myocytes and, also, the gap junctions are small.^8,9^ However, this is not the only reason: another reason is the nature of the connexin isoform expressed. The principal connexin in the working atrial and ventricular myocardium is the medium-conductance Cx43. The transitional tissue in the triangle of Koch in humans and rabbits does express Cx43, but the level is reduced as compared with the surrounding atrial muscle (Figure 1B).^3,5,10,11^ The compact node (CN) shows low expression of Cx43 in humans and animals; Figures. 1B, 2B, and 3B show that Cx43 is largely absent from the CN in humans^11^ and rabbits.^12^ Most studies have shown Cx43 mRNA and protein are also largely absent from the inferior nodal extension (INE) in humans^3^ and rabbits,^5,10,13^ and, as an example, Figure 1B shows the low level of Cx43 mRNA in the INE in humans.^3^ However, surprisingly, Hucker et al^11^ have reported high levels of Cx43 throughout the INE in humans. Cx43 is expressed in the penetrating bundle (PB), His bundle, and bundle branches in humans and rabbits at least (Figure 1B).^5,6,9,10,13^ The high-conductance Cx40 channel is known to be expressed in the working atrial (but not ventricular) myocardium. The pattern of expression of Cx40 shares some similarities with that of Cx43. Figure 1B shows that in humans, expression of Cx40 mRNA is lower in the transitional tissue and INE than in the atrial muscle, but not significantly so.^3^ Figures 1B and 3 show that, however, expression of Cx40 (at both mRNA and protein levels) is high in the CN and PB in humans and rats.^3^ Both bundle branches have been shown to have high levels of Cx40 in humans, rabbits, rats, and mice.^6,9^ There is more limited information regarding connexin expression in the Purkinje fibers, but Cx40 has been shown to be expressed highly in dog and rat Purkinje fibers.^14^ The pattern of expression of the medium- and high-conductance connexins Cx40 and Cx43 is compatible with the known fast conduction in the atrial muscle (∼80 cm/s), slower conduction in the INE (≤10 cm/s), and very fast conduction in the PB, His bundle, and Purkinje fibers (∼150 cm/s). What is responsible for electrical coupling (even though weak electrical coupling) in the AV node? It is assumed that the small-conductance Cx45 is responsible. Figure 1B shows a low level of expression of Cx45 mRNA in all tissues at the AV junction in humans.^3^ It has been shown that the ultra-small-conductance Cx30.2 is expressed in the AV node of mice.^15^ However, Figure 1B shows that the expression of Cx31.9 (human equivalent of Cx30.2) mRNA is very low in all tissues at the AV junction in humans. Cx31.9 is also undetected at the protein level in humans.^3,15^ It is unlikely, therefore, to be functionally important in humans.^3^

Gene knockout studies have been used to determine the functional significance of connexins in the mouse AV node and have demonstrated the importance of Cx30.2, Cx40, and Cx45 but not Cx43.^15^ Paradoxically, homozygous knockout of Cx30.2 in mice *accelerates* conduction in the AV node.^16^ It is possible that Cx30.2 *reduces* electrical coupling between nodal myocytes in the AV node by forming small-conductance heterotypic gap junctions with Cx40 or by a competitive effect of low-conductance channels on high-conductance channels. However, as already discussed, the human equivalent of Cx30.2 (Cx31.9) is not or poorly expressed.^15^ A homozygous Cx40 knockout mouse demonstrates a 20% increase in the PR interval with the slowing of conduction in both the AV node and the His-Purkinje system.^15^ The acceleration of conduction in the AV node caused by the knockout of Cx30.2 is normalized by knocking out Cx40 in addition to Cx30.2.^16^ It is possible that Cx30.2 is primarily expressed in the proximal part of the AV conduction axis and Cx40 in the distal part and the acceleration of conduction caused by the knockout of Cx30.2 from the proximal part equals the slowing of conduction caused by the knockout of Cx40 from the distal part.^16^ Homozygous knockout of either Cx43 or Cx45 is lethal during embryogenesis, which has a limited study of the AV node.^15^ A heterozygous Cx43 knockout mouse does not show any alteration in any electrocardiographic parameters, including the PR interval. The heterozygous knockout of Cx45, in addition to the homozygous knockout of Cx40, has been shown to lead to further increases in the PR interval beyond that caused by the knockout of Cx40 alone.^15^ As an aside, the PR interval as a measure of AV node conduction should be used with caution, because it also includes the conduction time from the sinus node to the AV node and also from the AV node to the ventricular muscle. For example, if there is pacemaker shift from the high crista terminalis to the low crista terminalis, there is a substantial shortening of the PR interval. It should be confirmed that AV conduction is affected (eg, by measuring AH interval). Furthermore, conduction in different parts of the AV node may be affected differentially; there is a paucity of data on the consequences of connexin manipulations on different parts of the AV node.

The AV node (or parts of it at least) not only lacks high-conductance connexins but also Na_v_1.5 as already mentioned and, consequently, the large and fast inward I_Na_.^17,18^ As a result, the action potential upstroke in the AV node is dependent on the smaller and slower inward Ca^2+^ current, which is the reason why the upstroke velocity of the action potential is low—one of the factors responsible for the slow conduction velocity in the AV node.^17,18^ We have used computer modeling to compare the roles of connexins and Na_v_1.5 (S Inada; data not published). In the first simulation, we used a model of a string of electrically coupled human atrial myocytes; each myocyte was represented by a biophysically detailed model of the human atrial action potential.^19^ The coupling conductance between the myocytes was reduced in line with the reduction in Cx40 and Cx43 mRNAs observed in the INE (compared to that in the atrial muscle) in humans (Figure 1B)^3^; it was assumed that the reduction in mRNA is translated into a similar reduction in protein and coupling conductance. This simulation suggests that the reduction in connexin expression in the INE will result in a ∼36% reduction in the conduction velocity (compared to that in the atrial muscle). In the second simulation, the model of the action potential of the human atrial myocyte was modified on the basis of mRNA levels of ion channels and so on in the INE (as a fraction of those in the atrial muscle)^3^; this resulted in an action potential typical of the INE. In particular, the action potential had a slow upstroke as a result of the low expression of Na_v_1.5. This simulation suggests that the reduction in Na_v_1.5 expression in the INE will result in a ∼77% reduction in the conduction velocity (compared to that in the atrial muscle). The final simulation suggests that, together, the decreases in Cx40, Cx43, and Na_v_1.5 expression will cause a ∼84% decrease in the conduction velocity. Although this suggests that Na_v_1.5 is more important than the connexins, this prediction has not been tested experimentally. The lack of Cx40, Cx43, and Na_v_1.5 in the AV node may not be coincidental: Shaw and Rudy^20^ have argued that if electrical coupling is weak, safe conduction of the action potential is dependent on Ca^2+^ current (rather than on I_Na_).

## Patterning of connexins underlies substrate of dual pathway nodal electrophysiology

Dual AV nodal electrophysiology refers to the concept of fast and slow pathways within the AV node. The fast and slow pathways are so called because they are the fastest and slowest pathways for the action potential through the AV node. Whereas the fast pathway is the normal route for action potential conduction, the slow pathway is important for the conduction of action potentials at short coupling intervals because the refractory period of the slow pathway is shorter than that of the fast pathway.^18^ The dual pathways are the substrate of AVNRT. During slow-fast AVNRT, there is antegrade conduction of the action potential along the slow pathway, retrograde conduction along the fast pathway, and then activation of the atrial muscle, after which there can be further cycles of reentry (online supplement Figures 1A and 1B and related text). In addition to slow-fast AVNRT, there is fast-slow AVNRT when the action potential circles in the opposite direction.^21^ Correlation of electrophysiology (optical mapping as well as intracellular and extracellular action potential recording) with the identification of the dual pathways (by immunolabeling of marker proteins as well as histology) has shown that the fast pathway corresponds to the transitional tissue, whereas the slow pathway corresponds to the INE (Figure 1A).^10,22,23^ The standard treatment of AVNRT is catheter ablation of the slow pathway: radiofrequency energy is applied to the coronary sinus ostium (site of INE), and this supports the hypothesis that the INE is the structure that underlies the slow pathway supporting AVNRT.^1^ The speed of conduction of the 2 pathways is consistent with connexin expression at the AV junction: the relatively high Cx40 and Cx43 expression in the transitional tissue is expected to lead to fast conduction, whereas the lower Cx40 and Cx43 expression in the INE is expected to lead to slower conduction.^12^ However, this is likely to be the only part of the reason for the difference in conduction velocity, because Na_v_1.5 expression in the transitional tissue is relatively high while it is low in the INE.^3^ By using a 3-dimensional anatomical model of the AV node incorporating the dual pathways^10^ together with biophysically detailed models of AV node action potentials,^18^ we are able to simulate AVNRT (online supplement Figure 1C and Movie 1). In the simulation, AVNRT is dependent on the poor electrical coupling (low coupling conductance) and consequent low conduction velocity in the slow pathway; in the simulation in online supplement Figure 1C, the ratio of longitudinal coupling conductance in the slow pathway, fast pathway, and atrial muscle was 29:160:625.

## Patterning of connexins reveals 2 nodal extensions

**Figure 4:**
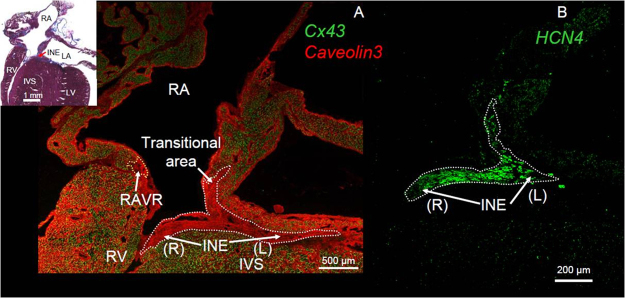
Two nodal extensions in the mouse heart. **A:** Immunolabeling of 4-chamber section through the mouse heart of connexin 43 (Cx43; green signal) and caveolin3 (myocyte marker; red signal). Inset: Sister section stained with Masson’s trichrome. **B:** Immunolabeling of sister section for HCN4 (marker of nodal tissue; green signal). From Yanni et al.^24^ INE = inferior nodal extension; IVS = interventricular septum; LA = left atrium; LV = left ventricle; RA = right atrium; RAVR = right atrioventricular ring; RV = right ventricle.

The patterning of connexins at the AV junction has resulted in some surprising findings. The first is that there are 2 nodal extensions. Figure 4 shows sister sections through the mouse heart immunolabeled for Cx43—caveolin3 (myocyte marker) and HCN4, the ion channel responsible for the funny current (nodal marker).^24^ It shows a left nodal extension as well as the well-known right nodal extension (INE) from the AV node; both nodal extensions are Cx43 negative. Right and left nodal extensions have also been demonstrated in rabbits and guinea pigs.^24,25^ The right nodal extension continues around the tricuspid valve annulus as the right AV ring, whereas the left nodal extension continues around the mitral valve annulus as the left AV ring.^24,26^ The right and left AV rings loop round the 2 valves and meet again to form the retroaortic node.^24^ The ring tissues are thought to arise from the embryological “primary myocardium” (AV canal in particular; see online supplement) that gives rise to the tissues of the cardiac conduction system, and in rats, they have a similar gene expression profile to the INE and CN.^6,24^ There are also reports of right and left nodal extensions in humans.^26,27^ Human studies have shown a limited left nodal extension that is shorter than the right nodal extension and not present in all subjects studied.^11,26,27^ Surprisingly, Hucker et al^11^ reported that whereas the left nodal extension is Cx43 negative, the right one is Cx43 positive; compare this with Figures. 1B and 4, which show the right nodal extension to be Cx43 negative in humans and mice. The significance of a left as well as a right nodal extension with regard to AVNRT is unclear. However, in a recent large case series of ablations for typical slow-fast AVNRT, 2% of the patients required a left-sided approach after an ablation from the right side had failed to successfully ablate the slow pathway.^28^ In atypical fast-slow AVNRT, there is a much greater need for left-sided ablation to eliminate slow pathway conduction.^29^ This suggests that the left nodal extension is functionally important.

## Patterning of connexins reveals longitudinal dissociation in PB

Another surprising finding revealed by the patterning of connexins concerns longitudinal dissociation in the PB. The PB is not a homogeneous cable. Striking images from immunohistochemistry have shown that although the upper part of the INE in rabbits^10^ and the CN in humans (Figure 3B)^9,11^ and rabbits (Figures 2B and 2C)^10,12^ is Cx43 negative, the lower part is Cx43 positive. The PB also shows longitudinal dissociation. In humans and rabbits, there are high levels of Cx43 in the lower part of the PB, whereas in rats and mice there are high levels of Cx40 in the lower part of the PB (Figure 3C).^6,11,12,30^ This has reinforced the concept of a “lower nodal bundle,” which was first suggested by a histological study of the rabbit AV node by Anderson et al.^31^Figure 2A shows that in the CN (area B), the myocytes are compactly arranged whereas in the lower nodal bundle (area C) the cells are loosely organized. It is tempting to speculate that the Cx43-positive right nodal extension reported by Hucker et al^11^ (see above) may in fact be the Cx43-positive lower nodal bundle. The origin of the longitudinal dissociation may lie in the embryological development of the heart: whereas the INE and CN are derived from the AV canal, the lower nodal myocytes in the PB (continuous with the His bundle) are derived from the ventricular myocardium.^30,32^ Rentschler et al^30^ showed that the inhibition of Notch signaling resulted in a loss of Cx30.2-expressing myocytes in the INE and CN but had no effect on the Cx40-expressing lower nodal myocytes in the PB.^30^

**Figure 5:**
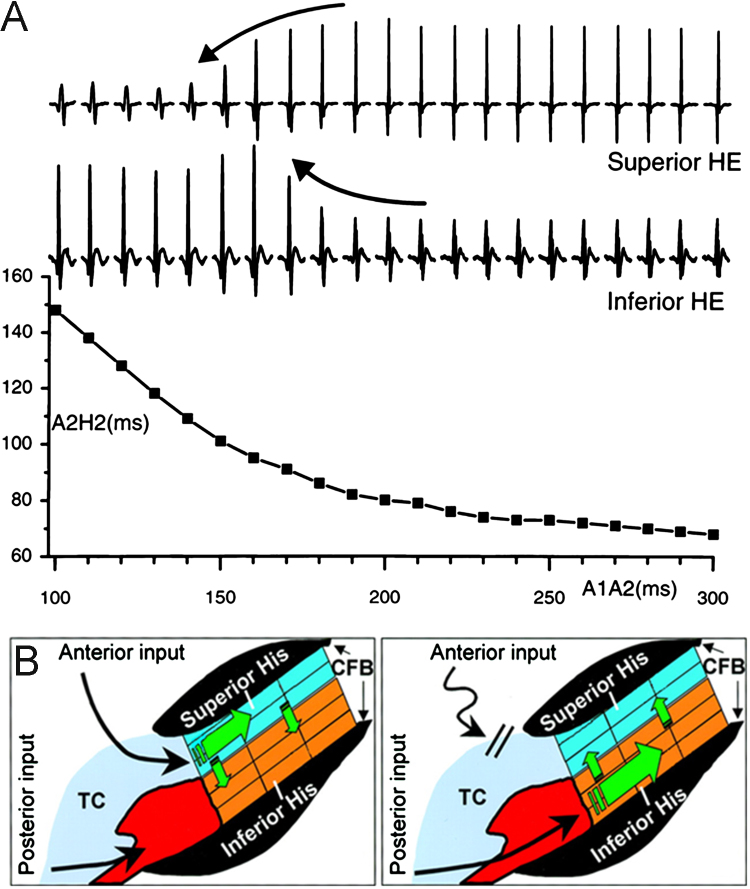
Demonstration of His alternans. **A:** Electrograms recorded from upper and lower parts of the penetrating bundle (PB) in rabbits in response to extrastimuli (delivered with the S1-S2 protocol; only response to S2 stimulus is shown in top panels) and the corresponding A2H2 interval plotted against the A1A2 interval. **B:** Schematic diagram showing longitudinal dissociation along PB such that the sequence of activation is dependent on how the PB is activated: via fast pathway or anterior input (left) or via slow pathway or posterior input (right). From Zhang et al.^33^ CFB = central fibrous body; HE = His electrode; TC = transitional cell.

**Figure 6:**
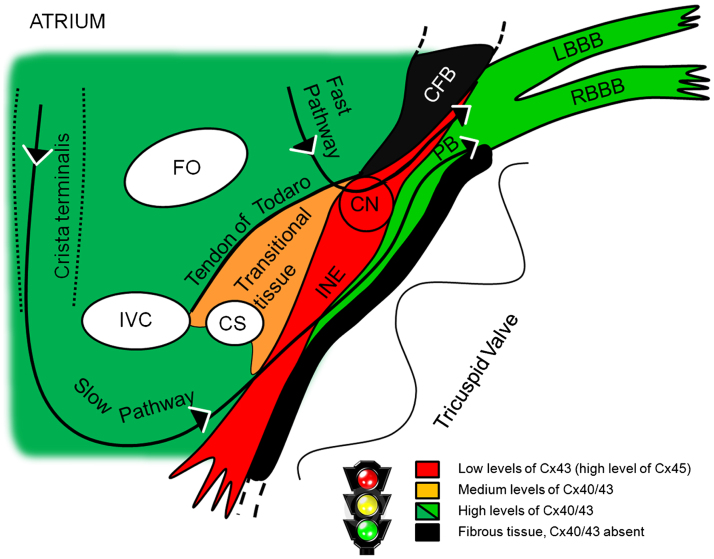
Summary of connexin expression at the atrioventricular (AV) junction. Traffic light colors summarize the level of expression of higher conductance connexin isoforms: connexin 40 (Cx40) and connexin 43 (Cx43). CN = compact node; CS = coronary sinus; FO = fossa ovalis; INE = inferior nodal extension; IVC = inferior vena cava; LBBB = left bundle branch; PB = penetrating bundle; RBBB = right bundle branch.

The activation pattern within the PB has been studied by using bipolar electrodes positioned over both the upper and lower parts (Figure 5A).^33^ When a premature beat is introduced by using an S1-S2 protocol, the fast pathway is activated with the subsequent activation pattern in the PB, showing activation occurs first in the upper part of the PB (Figure 5A).^33^ As the premature beat becomes earlier, there is a gradual transition from the PB being activated by the fast pathway to being activated by the slow pathway (Figure 5A).^33^ As this occurs, the sequence of activation within the PB changes such that the lower part of the PB is activated first until eventually action potential propagation blocks before reaching the PB with very premature stimuli (Figure 5A).^33^ Not only does the timing of the activation sequence change, but the amplitude of the recorded signal also alters, so that when the upper part of the PB is activated first the deflection is larger in the superior electrode and vice versa when the lower part is activated first (Figure 5A).^33^ This phenomenon has been termed “His alternans,” and it shows that the concept of dual pathway electrophysiology has to be extended to the PB.^33^ The His alternans shown in Figure 6A may be a consequence of the longer refractory period of the fast pathway if the fast pathway connects to the upper nodal bundle and the slow pathway (with a shorter refractory period) connects to the lower nodal bundle as suggested by Figure 5B. In this case, at short S1-S2 intervals, conduction through the fast pathway/upper nodal bundle will fail whereas conduction through the slow pathway/lower nodal bundle will persist (Figure 5C). However, this does not explain why the signal from the lower nodal bundle weakens at long S1-S2 intervals (Figure 5A). It is possible that at long S1-S2 intervals, the action potential traveling via the fast pathway retrogradely travels back up the slow pathway and blocks the slow pathway action potential.^18^Figure 5B suggests that the INE may connect to the lower nodal bundle. Hucker et al^25^ obtained evidence of such a connection in rabbits: they stimulated along the INE and observed activation of the slow pathway and PB. The time for conduction was dependent on the distance of the stimulus from the PB, and there was almost instantaneous activation of the PB when the stimulus was at the proximal (with respect to PB) end of the INE.^25^ This led Hucker et al^25^ to speculate that clinically it may be possible to pace within the region of the slow pathway to “bypass” the AV node and stimulate the PB directly.

It is plausible that the differential expression of connexins in the PB (Figure 3C) accounts for the functional differences between the upper and lower parts of the PB seen in His alternans. However, it is curious that the Cx43-positive fast pathway is contiguous with the upper part of the PB with low levels of Cx40/Cx43 and that the Cx43-negative slow pathway is contiguous with the lower part of the PB with high levels of Cx40/Cx43.

## Is connexin dysregulation responsible for AV node dysfunction?

In patients who had heart failure, there is an increase in the PR interval,^34^ which is associated with a high morbidity and mortality.^35^ We have reported a significant increase in the PR interval in a rat model of myocardial infarction and heart failure.^36^ If the increase in the PR interval is the result of impaired conduction through the AV node (this has not been demonstrated), it could be the result of a downregulation of connexin expression. Recently, we have observed a significant downregulation of Cx43 mRNA in a rabbit model of congestive heart failure (T Nikolaidou, X Cai, and G Hart, unpublished data). An increase in the PR interval (or AV conduction time) and incidence of first-degree heart block is also associated with aging and athletic training, and if this is the result of impaired conduction through the AV node, once more it could be the result of a downregulation of connexin expression.^34,37,38^ In addition to gap junctions, which provide electrical coupling of myocytes, there are several structures (tight junction is one) providing mechanical coupling of myocytes. There is crosstalk between tight and gap junctions: Lisewski et al^39^ showed that the inducible heart-specific knockout of a tight junction protein (Coxsackie virus-adenovirus receptor) leads to severe AV block and a downregulation of Cx43 and Cx45. Although there is no known equivalent human condition, this finding highlights a route of connexin regulation. Wolff-Parkinson-White syndrome is a heart condition in which there is an electrical pathway (accessory pathway), other than the AV node, connecting the atria and the ventricles. The condition can lead to episodes of reentrant tachycardia and is one of the most common causes of fast heart rate disorders in infants and children. The development of accessory pathways has been demonstrated in mice by using either activation of notch signaling or inactivation of Tbx2, indicating that dysregulation of the cell signaling pathways that are essential for the development of the AV canal and the AV node may be responsible for the Wolff-Parkinson-White syndrome.^30,40^ Tbx2 normally suppresses the expression of Cx40 and Cx34, and knockout of Tbx2 leads to the loss of the AV canal phenotype and high expression of Cx40 and Cx43 in an accessory pathway, allowing fast conduction between the atria and the ventricles.^40^

## Conclusions

Connexins are central to the functional role of the AV node. In Figure 6, we summarize the distribution of connexins in different tissues at the AV junction. The traffic light color scheme corresponds to high-, medium-, and low-strength electrical coupling, that is, fast, moderate, and slow conduction. The fast pathway corresponds to the transitional tissue and possibly the upper part of the PB. The slow pathway corresponds to the INE and possibly the lower part of the PB. It is not clear whether the CN is involved only in the fast pathway or is common to both pathways. However, in both pathways, the action potential will have to course through poorly coupled tissue lacking Cx40 and Cx43. As the PB extends distally into the bundle branches and Purkinje fibers, there is high expression of Cx40 and Cx43, facilitating rapid conduction.

## Figures and Tables

**Figure:**
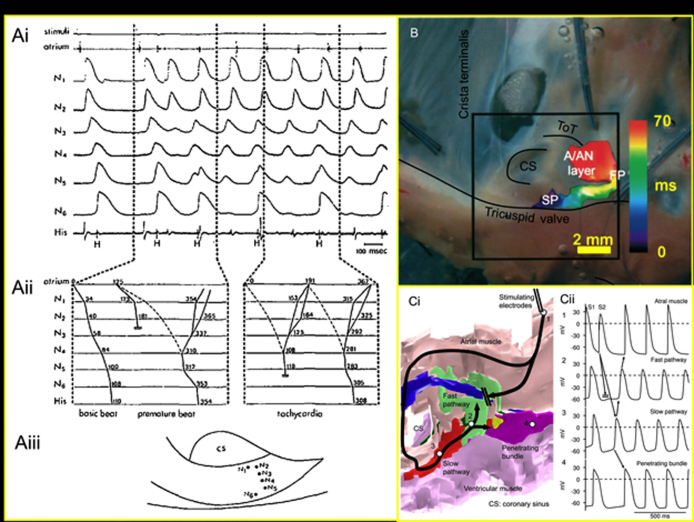


## References

[1] Markowitz S., Stein K., Mittal S., Lerman B., Mazgalev T., Tchou M.D. (2000). Dual atrionodal physiology in the human heart. Atrial-AV Nodal Electrophysiology: A View from the Millennium.

[2] Efimov I.R., Nikolski V.P., Rothenberg F. (2004). Structure-function relationship in the AV junction. Anat Rec A Discov Mol Cell Evol Biol.

[3] Greener I.D., Monfredi O., Inada S. (2011). Molecular architecture of the human specialised atrioventricular conduction axis. J Mol Cell Cardiol.

[4] de Groot J.R., Veenstra T., Verkerk A.O. (2003). Conduction slowing by the gap junctional uncoupler carbenoxolone. Cardiovasc Res.

[5] Greener I.D., Tellez J.O., Dobrzynski H. (2009). Ion channel transcript expression at the rabbit atrioventricular conduction axis. Circ Arrhythm Electrophysiol.

[6] Yoo S., Dobrzynski H., Fedorov V.V. (2006). Localization of Na^+^ channel isoforms at the atrioventricular junction and atrioventricular node in the rat. Circulation.

[7] Boyett M.R., Inada S., Yoo S. (2006). Connexins in the sinoatrial and atrioventricular nodes. Adv Cardiol.

[8] Pollack G.H. (1976). Intercellular coupling in the atrioventricular node and other tissues of the rabbit heart. J Physiol.

[9] Davis L.M., Rodefeld M.E., Green K., Beyer E.C., Saffitz J.E. (1995). Gap junction protein phenotypes of the human heart and conduction system. J Cardiovasc Electrophysiol.

[10] Li J., Greener I.D., Inada S. (2008). Computer three-dimensional reconstruction of the atrioventricular node. Circ Res.

[11] Hucker W.J., McCain M.L., Laughner J.I., Iaizzo P.A., Efimov I.R. (2008). Connexin 43 expression delineates two discrete pathways in the human atrioventricular junction. Anat Rec.

[12] Dobrzynski H., Nikolski V.P., Sambelashvili A.T. (2003). Site of origin and molecular substrate of atrioventricular junctional rhythm in the rabbit heart. Circ Res.

[13] Ko Y.S., Yeh H.I., Ko Y.L. (2004). Three-dimensional reconstruction of the rabbit atrioventricular conduction axis by combining histological, desmin, and connexin mapping data. Circulation.

[14] Kanter H.L., Laing J.G., Beau S.L., Beyer E.C., Saffitz J.E. (1993). Distinct patterns of connexin expression in canine Purkinje fibers and ventricular muscle. Circ Res.

[15] Jansen J.A., van Veen T.A., de Bakker J.M., van Rijen H.V. (2010). Cardiac connexins and impulse propagation. J Mol Cell Cardiol.

[16] Schrickel J.W., Kreuzberg M.M., Ghanem A. (2009). Normal impulse propagation in the atrioventricular conduction system of Cx30.2/Cx40 double deficient mice. J Mol Cell Cardiol.

[17] Hancox J.C., Yuill K.H., Mitcheson J.S., Convery M.K. (2003). Progress and gaps in understanding the electrophysiological properties of morphologically normal cells from the cardiac atrioventricular node. Int J Bifurcation Chaos.

[18] Inada S., Hancox J., Zhang H., Boyett M. (2009). One-dimensional mathematical model of the atrioventricular node including atrio-nodal, nodal, and nodal-his cells. Biophys J.

[19] Chandler N.J., Greener I.D., Tellez J.O. (2009). Molecular architecture of the human sinus node. Circulation.

[20] Shaw R.M., Rudy Y. (1997). Ionic mechanisms of propagation in cardiac tissue: roles of the sodium and L-type calcium currents during reduced excitability and decreased gap junction coupling. Circ Res.

[21] Nikolski V.P., Jones S.A., Lancaster M.K., Boyett M.R., Efimov I.R. (2003). Cx43 and dual-pathway electrophysiology of the atrioventricular node and atrioventricular nodal reentry. Circ Res.

[22] Janse M., Van Capelle F., Freud G., Durrer D. (1971). Circus movement within the AV node as a basis for supraventricular tachycardia as shown by multiple microelectrode recording in the isolated rabbit heart. Circ Res.

[23] Loh P., Ho S.Y., Kawara T. (2003). Reentrant circuits in the canine atrioventricular node during atrial and ventricular echoes: electrophysiological and histological correlation. Circulation.

[24] Yanni J., Boyett M.R., Anderson R.H., Dobrzynski H. (2009). The extent of the specialized atrioventricular ring tissues. Heart Rhythm.

[25] Hucker W.J., Sharma V., Nikolski V.P., Efimov I.R. (2007). Atrioventricular conduction with and without AV nodal delay: two pathways to the bundle of His in the rabbit heart. Am J Physiol Heart Circ Physiol.

[26] Waki K., Kim J.S., Becker A.E. (2000). Morphology of the human atrioventricular node is age dependent: a feature of potential clinical significance. J Cardiovasc Electrophysiol.

[27] Inoue S., Becker A.E. (1998). Posterior extensions of the human compact atrioventricular node: a neglected anatomic feature of potential clinical significance. Circulation.

[28] Giazitzoglou E., Korovesis S., Kokladi M., Venetsanakos I., Paxinos G., Katritsis D.G. (2010). Slow-pathway ablation for atrioventricular nodal re-entrant tachycardia with no risk of atrioventricular block. Hellenic J Cardiol.

[29] Otomo K., Okamura H., Noda T. (2006). “Left-variant” atypical atrioventricular nodal reentrant tachycardia: electrophysiological characteristics and effect of slow pathway ablation within coronary sinus. J Cardiovasc Electrophysiol.

[30] Rentschler S., Harris B.S., Kuznekoff L. (2011). Notch signaling regulates murine atrioventricular conduction and the formation of accessory pathways. J Clin Invest.

[31] Anderson R.H., Janse M.J., van Capelle F.J., Billette J., Becker A.E., Durrer D. (1974). A combined morphological and electrophysiological study of the atrioventricular node of the rabbit heart. Circ Res.

[32] Aanhaanen W.T.J., Mommersteeg M.T.M., Norden J. (2010). Developmental origin, growth, and three-dimensional architecture of the atrioventricular conduction axis of the mouse heart. Circ Res.

[33] Zhang Y., Bharati S., Mowrey K.A., Zhuang S., Tchou P.J., Mazgalev T.N. (2001). His electrogram alternans reveal dual-wavefront inputs into and longitudinal dissociation within the bundle of His. Circulation.

[34] Crisel R.K., Farzaneh-Far R., Na B., Whooley M.A. (2011). First-degree atrioventricular block is associated with heart failure and death in persons with stable coronary artery disease: data from the Heart and Soul Study. Eur Heart J.

[35] Gervais R., Leclercq C., Shankar A. (2009). Surface electrocardiogram to predict outcome in candidates for cardiac resynchronization therapy: a sub-analysis of the CARE-HF trial. Eur J Heart Fail.

[36] Yanni J., Tellez J.O., Mączewski M. (2011). Changes in ion channel gene expression underlying heart failure-induced sinoatrial node dysfunction. Circ Heart Fail.

[37] Schmidlin O., Bharati S., Lev M., Schwartz J. (1992). Effects of physiological aging on cardiac electrophysiology in perfused Fischer 344 rat hearts. Am J Physiol Heart Circ Physiol.

[38] Stein R., Medeiros C.M., Rosito G.A., Zimerman L.I., Ribeiro J.P. (2002). Intrinsic sinus and atrioventricular node electrophysiologic adaptations in endurance athletes. J Am Coll Cardiol.

[39] Lisewski U., Shi Y., Wrackmeyer U. (2008). The tight junction protein CAR regulates cardiac conduction and cell-cell communication. J Exp Med.

[40] Aanhaanen W.T.J., Boukens B.J.D., Sizarov A. (2011). Defective Tbx2-dependent patterning of the atrioventricular canal myocardium causes accessory pathway formation in mice. J Clin Invest.

