# Supplementary material for: Connexins and the atrioventricular node
Source: Heart Rhythm. 2013 Feb;10(2):297–304. doi: 10.1016/j.hrthm.2012.10.020 (PMC3572393; doi:10.1016/j.hrthm.2012.10.020)
Supplement: Supplementary file 1 — Supplementary Material [file mmc1.doc]

# Online Supplement

# Connexins and the atrioventricular node

# Ian P. Temple, Shin Inada, Halina Dobrzynski and Mark R. Boyett

Electrical coupling between cardiac myocytes is provided by gap junctions made up of connexins. *Heart Rhythm* has recently published an excellent series of ‘Viewpoint’ articles on connexins and, for an introduction to connexins,[^1^](#_ENREF_1) and information on arrhythmogenic remodelling of connexins in disease states,[^2^](#_ENREF_2) the possibility that connexins couple myocytes and fibroblasts,[^3^](#_ENREF_3) the new concept of the perinexus,[^4^](#_ENREF_4) and regulation of connexins by intracellular signalling pathways and crosstalk between connexins and desmosomes,[^5^](#_ENREF_5) the reader is referred to the series.

# History, embryology and anatomy of the atrioventricular (AV) node

Usually the discovery of the AV node is attributed to a young Japanese investigator, Sunao Tawara, working under the tutelage of Professor Luwig Aschoff at the University of Marburg in Germany. In 1906, Tawara published his anatomical studies of the AV node as a monograph, which was reprinted in 2000.[^6^](#_ENREF_6) However, much earlier, Walter Gaskell was working on the tortoise heart at the Physiological Laboratory in Cambridge in England.[^7^](#_ENREF_7),[^8^](#_ENREF_8) In 1883, Gaskell wrote that “a peristaltic wave [the action potential], … at the auriculo-ventricular junction passes through a region of diminished conductivity” and then “increasing impairment of the tissue, produces first a commencing block, i.e. a simple delay in the rate of passage of each contraction wave, then a partial block, so that only every second contraction passes, and finally a complete block, in which no contractions pass”.[^7^](#_ENREF_7) These are remarkable first insights into the AV node. Slow conduction across the AV node is thought in part to be due poor electrical between cells at the AV node since. This was first demonstrated in the pioneering work of Pollack in 1976.[^9^](#_ENREF_9) Pollack[^9^](#_ENREF_9) injected fluorescein intracellularly by micro-iontophoresis into the rabbit AV node and tracked its diffusion and showed that the rate of passage of dye between nodal myocytes is at least three orders of magnitude lower than between myocytes of the other tissues studied.

In the embryo, the heart is initially a simple tubular structure composed of ‘primary myocardium’ with a phenotype (including slow conduction, pacemaking and poor contraction) that resembles that of nodal tissues.[^10^](#_ENREF_10) Later the developing atrial and ventricular chambers, composed of ‘working myocardium’, bud off from the tube of primary myocardium.[^10^](#_ENREF_10) The working myocardium is distinct from the primary myocardium in a number of ways, including the possession of gap junctions made up of Cx40 and Cx43, which permit rapid conduction.[^10^](#_ENREF_10) Parts of the primary myocardium retain their initial embryonic phenotype and are destined to form the cardiac nodes and AV conduction axis in the adult heart.[^10^](#_ENREF_10) One part, the AV canal, is destined to form the AV node (as well as rings of nodal-like myocytes encircling the tricuspid and mitral valves, the so-called AV rings). All of this is controlled by transcription factors as reviewed elsewhere. For example, Tbx2, Tbx3 and Msx2 are selectively expressed in the AV canal and together they suppress the expression of Cx40 and Cx43.[^10^](#_ENREF_10) Inactivation of Tbx2 and Tbx3 causes the loss of the AV canal phenotype.[^10^](#_ENREF_10)

When discussing the anatomy of the AV node in the adult heart, it is important to be aware of ambiguity with regards to the precise naming of the different regions.[^13^](#_ENREF_13) The AV node is contained within the triangle of Koch, which is defined as the region between the tendon of Todaro, ostium of the coronary sinus and the tricuspid valve annulus at the base of the right atrium (Fig. 1A). In this region, a tract of specialised nodal and nodal-like myocytes extends from the right atrium through the ring of fibrous tissue (separating the upper and lower chambers) into the ventricles, where it emerges as the His bundle, which then bifurcates into the left and right bundle branches (Fig. 1A). It has been proposed that the term ‘AV node’ should be reserved for the nodal tissue that is not enclosed within the fibrous tissue of the central fibrous body, whilst the term ‘penetrating bundle’ should be reserved for the portion of the conduction system that is enclosed (Fig. 1A).[^14^](#_ENREF_14) Within the AV node, there is a region of tightly-packed nodal myocytes termed the compact node (CN; Fig. 1A).[^15-17^](#_ENREF_15) The CN in the rabbit is shown in Fig. 2A and in the human in Fig. 3B. Extending from this is the inferior nodal extension (INE), which has also been (incorrectly) termed the posterior nodal extension (Fig 1A). ‘Inferior’ refers to the orientation of the nodal extension in the heart in the upright human (Fig. 1A).[^13^](#_ENREF_13) The INE is largely made up of small loosely-packed nodal myocytes.[^16^](#_ENREF_16) The INE and CN are in contact with loosely-packed transitional myocytes, which share characteristics of both nodal myocytes and working atrial myocytes (Fig. 1A). The transitional myocytes are in direct contact with the atrial muscle (Fig. 1A).[^17^](#_ENREF_17)

# AV nodal reentrant tachycardia (AVNRT)

The concept of dual AV nodal electrophysiology refers to the dual inputs (fast and slow pathways) into the AV node shown in Fig. 1A. This concept is supported by a wealth of both clinical^e.g.^[^18^](#_ENREF_18) and experimental^e.g.^[^19^](#_ENREF_19) data. The possibility of dual AV nodal electrophysiology as the substrate for reentrant arrhythmias dates back to Mines in 1913.[^20^](#_ENREF_20) Online supplement Fig. 1 shows three examples of AVNRT. Online supplement Fig. 1A shows early work using microelectrodes from 1971:[^19^](#_ENREF_19) following S1 stimuli, activation proceeded from N_1_ to N_6_ along the fast pathway. Following a S2 stimulus, the premature action potential proceeded from N_1_ to N_3_ and then blocked. Meanwhile the premature beat must have entered the AV node via the slow pathway arriving at site N_4_ first. From site N4, the premature beat proceeded retrogradely along the fast pathway from site N_4_ to N_1_ and then into the atrium as an atrial echo beat. This then subsequently reentered the slow pathway resulting in AVNRT. Recordings were not made from the slow pathway in this experiment. Supplement Fig. 1B shows later work using optical mapping from 2003:[^21^](#_ENREF_21) antegrade conduction along the slow pathway was followed by retrograde conduction along the fast pathway and finally breakout into the atrium. Finally, online supplement Fig. 1C shows a recent unpublished computer modelling study investigating typical slow-fast AVNRT (S. Inada; unpublished data): conduction into the fast pathway was blocked as a result of its relatively long refractory period. There was antegrade conduction along the slow pathway followed by retrograde conduction along the fast pathway and finally breakout into the atrium. The movie in the online supplement shows an animation of this simulation. These studies show striking consistency with the slow spread of electrical activity in the antegrade direction along the slow pathway before breakout into the atrium via retrograde conduction along the fast pathway.[^21^](#_ENREF_21) This allows the slow pathway to be reactivated by the atrium setting up a stable reentrant circuit, which underlies AVNRT.

# Supplement references

**1.** Smyth JW, Shaw RM: The gap junction life cycle. Heart Rhythm 2012; 9:151-153.

**2.** Wit AL, Peters NS: The role of gap junctions in the arrhythmias of ischemia and infarction. Heart Rhythm 2012; 9:308-311.

**3.** Kohl P, Camelliti P: Fibroblast-myocyte connections in the heart. Heart Rhythm 2012; 9:461-464.

**4.** Rhett JM, Gourdie RG: The perinexus: A new feature of Cx43 gap junction organization. Heart Rhythm 2012; 9:619-623.

**5.** Delmar M, Liang FX: Connexin43 and the regulation of intercalated disc function. Heart Rhythm 2012; 9:835-838.

**6.** Tawara S, Suma K, Shimada M, Corporation E. *The conduction system of the mammalian heart: an anatomico-histological study of the atrioventricular bundle and the Purkinje fibers*: Imperial College press; 2000.

**7.** Gaskell WH: On the innervation of the heart, with especial reference to the heart of the tortoise. J Physiol 1883; 4:43-230 214.

**8.** Silverman ME, Grove D, Upshaw Jr CB: Why does the heart beat? Circulation 2006; 113:2775-2781.

**9.** Pollack GH: Intercellular coupling in the atrioventricular node and other tissues of the rabbit heart. J Physiol 1976; 255:275-298.

**10.** Christoffels VM, Smits GJ, Kispert A, Moorman AFM: Development of the pacemaker tissues of the heart. Circ Res 2010; 106:240-254.

**11.** Yanni J, Boyett MR, Anderson RH, Dobrzynski H: The extent of the specialized atrioventricular ring tissues. Heart Rhythm 2009; 6:672-680.

**12.** Hatcher CJ, Basson CT: Specification of the cardiac conduction system by transcription factors. Circ Res 2009; 105:620-630.

**13.** Cosio FG, Anderson RH, Kuck KH, et al.: Living anatomy of the atrioventricular junctions. A guide to electrophysiologic mapping. A Consensus Statement from the Cardiac Nomenclature Study Group, Working Group of Arrhythmias, European Society of Cardiology, and the Task Force on Cardiac Nomenclature from NASPE. Circulation 1999; 100:e31-37.

**14.** Zhang Y, Bharati S, Mowrey KA, Zhuang S, Tchou PJ, Mazgalev TN: His electrogram alternans reveal dual-wavefront inputs into and longitudinal dissociation within the bundle of His. Circulation 2001; 104:832-838.

**15.** Anderson RH, Janse MJ, van Capelle FJ, Billette J, Becker AE, Durrer D: A combined morphological and electrophysiological study of the atrioventricular node of the rabbit heart. Circ Res 1974; 35:909-922.

**16.** Li J, Greener ID, Inada S, et al.: Computer three-dimensional reconstruction of the atrioventricular node. Circ Res 2008; 102:975-985.

**17.** Mazgalev TN, Ho SY, Anderson RH: Anatomic-electrophysiological correlations concerning the pathways for atrioventricular conduction. Circulation 2001; 103:2660-2667.

**18.** Markowitz S, Stein K, Mittal S, Lerman B. Dual atrionodal physiology in the human heart. In: Mazgalev T, Tchou, MD, ed. *Atrial-AV nodal electrophysiology: a view from the millennium.* Armonk, New York: Futura Publishing Company; 2000:353-370.

**19.** Janse M, Van Capelle F, Freud G, Durrer D: Circus movement within the AV node as a basis for supraventricular tachycardia as shown by multiple microelectrode recording in the isolated rabbit heart. Circ Res 1971; 28:403-414.

**20.** Mines GR: On dynamic equilibrium in the heart. J Physiol 1913; 46:349-383.

**21.** Nikolski VP, Jones SA, Lancaster MK, Boyett MR, Efimov IR: Cx43 and dual-pathway electrophysiology of the atrioventricular node and atrioventricular nodal reentry. Circ Res 2003; 92:469-475.

**22.** Inada S, Hancox J, Zhang H, Boyett M: One-dimensional mathematical model of the atrioventricular node including atrio-nodal, nodal, and nodal-his cells. Biophys J 2009; 97:2117-2127.

**Online movie**

Simulation of slow-fast reentry using electrophysiologically-detailed action potential model. The atrial septum was stimulated. Response to S1 (basic) and S2 (premature) stimuli are shown. S1-S1 interval was 350 ms, and S1-S2 interval was 130 ms. After basic stimuli were applied (at 5 and 355 ms), the action potential propagated from the atrium to the bundle of His via the fast pathway. After a premature stimulus was applied (at 485 ms), slow-fast reentry occurred. Three reentrant beats were observed and finally reentry self-terminated.

**
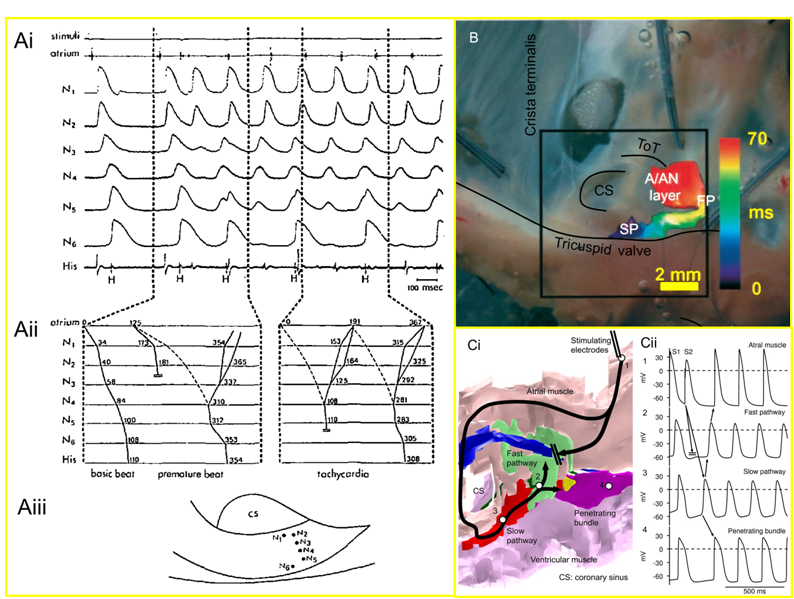
**

**Online supplement Fig. 1. AVNRT.** A, slow-fast AVNRT in rabbit AV node preparation induced by S1-S2 stimulation. From top to bottom: S1 and S2 stimuli; extracellular recording from atrial muscle; intracellular action potential recordings from sites N_1_ to N_6_; extracellular recording from His bundle; ladder diagram demonstrating sequence of activation; schematic diagram of preparation demonstrating location of intracellular recording sites N_1_ to N_6_. Coronary sinus (CS). From Janse et al.[^19^](#_ENREF_19) B, optical mapping of conduction during slow-fast AVNRT in rabbit AV junction preparation. Activation map (colour coded; calibration bar shown) in region of recording (box) is shown superimposed on photograph of preparation. Modified from Nikolski et al.[^21^](#_ENREF_21) Coronary sinus (CS), fast pathway (FP), slow pathway (SP) ,Tendon of Todaro (ToT). C, simulated slow-fast AVNRT induced by S1-S2 stimulation (S1-S2 interval, 130 ms). 3D anatomical model of rabbit AV node[^16^](#_ENREF_16) (Ci) was used in conjunction with biophysically-detailed models of rabbit AV node action potentials.[^22^](#_ENREF_22) Preparation was stimulated at top right. Conduction sequence following S2 stimulus is shown by arrows and conduction block is indicated by pair of parallel lines. Selected action potentials from atrial muscle, fast pathway, slow pathway and bundle of His (sites 1-4 on left in Ci) are shown on right (Cii). Conduction sequence is shown by arrows and conduction block is indicated by a pair of parallel lines. S1 and S2 beats were followed by a series of reentry beats. From S. Inada (unpublished data).
